# Efficacy and Safety of the Preserflo^®^ Microshunt as a Standalone Procedure Versus Its Combination with Phacoemulsification

**DOI:** 10.3390/jcm15103583

**Published:** 2026-05-07

**Authors:** Sieta Gassama, Paul Bastelica, Mathilde Huard, Karine Lagrené, Zohra Brouk, Esther Blumen-Ohana, Emmanuelle Brasnu-De-Cenival, Juliette Buffault, Pascale Hamard, Jean-Philippe Nordmann, Antoine Rousseau, Christophe Baudouin, Antoine Labbé

**Affiliations:** 1 Department of Ophthalmology, Quinze-Vingts National Ophthalmology Hospital, IHU FOReSight, 75012 Paris, Francejbuffault@15-20.fr (J.B.);; 2 Department of Ophthalmology, Ambroise Paré Hospital, AP-HP, University of Versailles Saint-Quentin-en-Yvelines, 92100 Boulogne-Billancourt, France; 3 Quinze-Vingts National Ophthalmology Hospital, IHU FOReSight, INSERM-DGOS CIC 1423, 75571 Paris, France; 4 Department of Ophthalmology, Assistance Publique Hôpitaux de Paris, Centre de Référence Maladies Rares en Ophtalmologie (OPHTARA), 94275 Le Kremlin-Bicêtre, France

**Keywords:** glaucoma, combined glaucoma surgery, Preserflo Microshunt, phacoemulsification, MIBS, intraocular pressure

## Abstract

**Background/Objectives**: Glaucoma is a leading cause of irreversible blindness, and lowering intraocular pressure (IOP) is the only proven strategy to slow disease progression. The Preserflo^®^ Microshunt (PMS) is a minimally invasive subconjunctival drainage device used to treat uncontrolled glaucoma. Given that cataracts and glaucoma often coexist, this study aimed to compare the 12-month efficacy and safety of PMS implanted as a standalone procedure versus combined with phacoemulsification (PCE). **Methods**: This single-center retrospective case–control study included 104 eyes (26 PMS + PCE; 78 standalone PMS) from patients treated between 2019 and 2023. Controls were matched 3:1 to cases on sex, age, glaucoma severity, baseline IOP, and number of prior glaucoma surgeries. Success at 12 months was defined as absolute (IOP ≤ 21 mmHg without medications) or qualified (IOP ≤ 21 mmHg with the same or fewer medications). Secondary outcomes included changes in IOP, medication burden, and safety profiles. **Results**: At 12 months, absolute success was achieved in 23.1% of the PMS + PCE group versus 43.6% in the standalone PMS group (*p* = 0.13). However, the Kaplan–Meier survival probability of absolute success was significantly higher for standalone PMS (75.2% vs. 40.2%, *p* = 0.0028). Qualified success rates were comparable (53.9% vs. 70.5%, *p* = 0.30). Both groups showed similar reductions in mean IOP and medication counts. Although safety profiles were comparable, the mean time to first bleb revision (*p* = 0.031) and first needling (*p* = 0.015) was significantly shorter in the combined group. **Conclusions**: Both standalone and combined PMS procedures are effective and safe in achieving sustained IOP reduction. Although standalone implantation appears to be associated with higher medication-free success rates, combined surgery remains a reasonable option for patients with coexisting cataract, despite a tendency toward earlier bleb fibrosis likely related to the inflammatory response induced by PCE.

## 1. Introduction

Glaucoma is a chronic and progressive optic neuropathy characterized by accelerated degeneration of retinal ganglion cells [1]. It remains a leading cause of irreversible blindness worldwide, with an estimated 64.3 million individuals affected in 2016 and projections indicating an increase to 111.8 million by 2040 due to population aging [2].

Lowering intraocular pressure (IOP) is currently the only therapeutic strategy proven to slow disease progression. IOP reduction may be achieved through medical therapy with topical or systemic hypotensive agents, laser procedures, or surgery. Surgical interventions are considered when optic neuropathy progresses despite maximally tolerated medical therapy. Among conventional filtering procedures, trabeculectomy is considered the gold standard, remains the most commonly performed, and has demonstrated long-term efficacy in glaucoma management [3,4]. More recently, a range of less invasive filtering procedures—collectively referred to as Minimally Invasive Bleb Surgery (MIBS)—has emerged as an alternative to conventional filtering procedures. These techniques are designed to reduce the incidence of complications associated with traditional filtering surgery, particularly postoperative hypotony, while promoting more rapid visual rehabilitation and providing a superior overall safety profile [5].

The Preserflo Microshunt (PMS) (Santen, Osaka, Japan) is an *ab externo* subconjunctival drainage device that diverts aqueous humor to the subconjunctival space via formation of a filtration bleb (MIBS). It consists of a tube composed of a biologically inert polymer (Poly-SIBS: poly-styrene-block-isobutylene-block-styrene) with a 70 μm lumen and an overall length of 8.5 mm, directing aqueous outflow to the posterior sub-Tenon space. By design, and according to Poiseuille’s law, the device dimensions are intended to provide intrinsic flow resistance to reduce the risk of postoperative ocular hypotony [6]. Efficacy has been demonstrated in moderate to advanced glaucoma, although outcomes may remain inferior to those achieved with trabeculectomy [7].

Because both cataracts and glaucoma are age-related conditions, their coexistence is common in older adults. In such cases, combined phacoemulsification (PCE) and filtering surgery may be considered an option to provide immediate visual rehabilitation while concurrently addressing IOP control, although a stepwise approach remains a frequent alternative. However, conventional filtering procedures may be less effective when performed concurrently with cataract extraction [8,9,10,11,12,13]. Conversely, newer subconjunctival drainage procedures such as PMS avoid scleral flap dissection and iridectomy, potentially limiting the release of pro-fibrotic mediators in the periconjunctival space relative to conventional filtering surgery [14,15]. As a result, conjunctival wound-healing dynamics may differ by surgical approach and by the presence or absence of an implant, limiting the applicability of conclusions drawn from studies of combined *versus* standalone conventional filtering procedures to modern subconjunctival devices.

Evidence regarding the efficacy and safety of PMS combined with PCE remains limited, with only two comparative studies to date, both with limited sample sizes and without matching for key confounders such as age, glaucoma severity, and prior glaucoma surgery [16,17]. These studies reported no significant differences in efficacy or safety between standalone PMS implantation and PMS combined with PCE, but their designs limit inference. The objective of the present study was to compare the 12-month efficacy and safety of standalone PMS implantation in pseudophakic patients versus PMS combined with PCE in a matched controlled study.

## 2. Materials and Methods

### 2.1. Study Design

This retrospective case–control study was conducted at the “Hôpital National de la Vision des Quinze-Vingts”, Paris, France, and reports 12-month outcomes for a cohort of patients with glaucoma uncontrolled with maximally tolerated medical therapy who underwent PMS surgery either as a standalone procedure or combined with PCE between January 2019 and December 2023. The study was conducted in accordance with the principles of the Declaration of Helsinki. All measurements were performed as part of routine care, and all data were collected anonymously. A non-objection form was mailed to the patients, and none of them objected to the use of their anonymized medical data. This study was approved by the Ethics Committee of the French Society of Ophthalmology (IRB00008855 Société Française d’Ophtalmologie IRB#1).

### 2.2. Patients

Consecutive adult patients (>18 years) with primary or secondary glaucoma who underwent combined PMS with PCE (PMS + PCE) between January 2019 and December 2023 were included. Consecutive pseudophakic patients who underwent the same PMS procedure as standalone surgery (PMS) were identified as potential controls and matched 3:1 to the PMS + PCE group based on five variables: sex, age, glaucoma severity (Hodapp–Anderson–Parrish classification) [18], baseline IOP, and number of prior glaucoma surgeries (including trabeculectomy, deep non-penetrating sclerectomy, Xen^®^ Gel Stent 45 [AbbVie, Chicago, IL, USA], drainage devices, and cyclodestructive procedures). Phakic patients were specifically excluded from the standalone group to eliminate the confounding hypotensive effect of lens extraction, allowing a more accurate comparison of the efficacy of the PMS implant between the two groups. Additional exclusion criteria were a baseline visual acuity of no light perception and the absence of intraoperative mitomycin C (MMC) use.

### 2.3. Surgical Technique

The PMS was provided in a sterile kit including a 3 mm scleral marker, a 1 mm triangular-blade knife, a 23-gauge cannula, a marker pen, and a 25-gauge needle. All procedures were performed under local anesthesia (sub-Tenon or subconjunctival injection of lidocaine) by seven experienced glaucoma surgeons. A limbal peritomy approximately 3–4 mm in length was fashioned in the superonasal or superotemporal quadrant, followed by posterior conjunctivo-Tenon dissection and light cautery; mitomycin C (MMC, 0.2 or 0.4 mg/mL) was then applied to the subconjunctival space using soaked sponges for 2 min. After marking the sclera 3 mm posterior to the limbus, a 1 mm superficial scleral pocket was created with the calibrated knife; a 25-gauge needle was then passed through the scleral pocket into the anterior chamber and retracted to create a scleral tunnel. The PMS was inserted bevel-up through the tunnel with the fins seated within the scleral pocket; a 23-gauge cannula was used to irrigate the device, and aqueous egress was confirmed by visualization of droplets at the distal tip. The distal tip was then advanced beneath Tenon’s capsule, and Tenon and conjunctiva were closed watertight as separate layers using either 8-0 or 10-0 absorbable sutures.

In combined procedures, standard PCE was performed after preparation of the filtration site and before PMS implantation, as described above. A 1.8–2.2 mm main corneal incision and a 1.5 mm side port were created, followed by injection of a viscoelastic agent; a manual capsulorrhexis and gentle hydrodissection were performed. PCE was carried out using a Centurion system (Alcon, Geneva, Switzerland) or Stellaris system (Bausch & Lomb, Rochester, NY, USA) with a divide-and-conquer technique; a posterior chamber intraocular lens was implanted in the capsular bag. The viscoelastic was thoroughly aspirated, corneal wounds were hydrated for watertight sealing, and PMS implantation was subsequently completed.

### 2.4. Postoperative Management

All antiglaucoma medications (topical and systemic) were discontinued immediately after surgery. All patients received topical dexamethasone, tobramycin, and artificial tears for 1 month to 3 months, as per clinical judgment. Patients were examined at a minimum on day 1, day 7, and months 1, 3, 6, and 12, including IOP measurement by Goldmann applanation tonometry, slit-lamp assessment with careful evaluation of the filtration bleb before and after fluorescein instillation, and fundus examination by indirect ophthalmoscopy. Reintroduction of hypotensive therapy, needling, or surgical bleb revision was performed at the surgeon’s discretion whenever IOP control was deemed insufficient. Needling was performed in clinic with a 25- or 30-gauge needle with an adjunctive subconjunctival injection of 5-fluorouracil (0.2 mL of a 25 mg/mL solution), whereas bleb revisions were performed in the operating room following the initial PMS procedure and consisted of subconjunctival and sub-Tenon fibrosis dissection, tube-tip mobilization, and re-application of MMC for 2 to 3 min.

### 2.5. Data Collection

Study data were collected and managed using REDCap (version 15.5.17) electronic data capture tools hosted at the Quinze-Vingts Hospital [19,20]. Baseline socio-demographic, clinical, and ancillary data were retrospectively collected from medical records and included: age, sex, type of glaucoma, best-corrected visual acuity (BCVA), refraction, IOP by Goldmann applanation tonometry, number of hypotensive medications, vertical cup-to-disc (C/D) ratio, and visual field mean deviation (SITA-Standard algorithm, Humphrey 24-2; Humphrey Visual Field Analyzer, Zeiss, Oberkochen, Germany). Prior selective laser trabeculoplasty (SLT) and the number and type of previous glaucoma procedures (trabeculectomy, deep non-penetrating sclerectomy, Xen^®^ Gel Stent 45, drainage devices, and diode laser cyclodestruction) were also recorded. Postoperative BCVA, refraction, IOP by Goldmann tonometry, and number of hypotensive medications were collected on day 7 and months 1, 3, 6, 12, 18, 24, and 36. Complications during follow-up were also assessed. Transient hypotony was defined as an IOP < 6 mmHg lasting less than 1 month, and chronic hypotony as an IOP < 6 mmHg measured at two successive visits 3 months apart. A decrease in at least two lines of visual acuity on the Monoyer chart was considered a severe complication.

### 2.6. Outcomes

The primary outcome was the proportion of eyes achieving “absolute” or “qualified” surgical success at 12 months. Surgical success assessed 12 months after surgery was defined as an IOP ≤ 21 mmHg without antiglaucoma medications (absolute success) or with the same number or fewer medications (qualified success). Therapeutic failure was defined as IOP > 21 mmHg at 12 months, the need for additional glaucoma surgery during follow-up (excluding bleb needling or surgical revision), or the occurrence of chronic hypotony and/or loss of light perception. Needling and bleb revision were not considered failures. Secondary outcomes included longitudinal changes in IOP and number of hypotensive medications, postoperative complications, and rates of needling and bleb revision.

### 2.7. Statistical Analysis

All analyses were performed using R (version 4.4.1; R Foundation for Statistical Computing, Vienna, Austria). Both eyes of a given patient could be included if they independently met the inclusion criteria. Each eye was treated as an independent statistical unit. This approach was justified by the relatively low proportion of bilateral cases and by the distinct clinical profiles frequently observed between fellow eyes of the same patient. Consequently, no specific adjustment for inter-eye correlation was performed. Continuous variables are presented as mean ± standard deviation, and categorical variables are presented as counts and percentages. Between-group comparisons for continuous variables were performed using the Mann–Whitney test for two unmatched groups or one-way analysis of variance for three groups, as appropriate; categorical variables were compared using the χ^2^ test or Fisher’s exact test, as indicated. Matching between the treated group (PMS + PCE) and the control group (PMS) was performed using optimal pair matching with a 3:1 ratio via the MatchIt package [21,22]. Event-free survival was compared using Kaplan–Meier curves and the log-rank test; two-sided *p*-values < 0.05 were considered statistically significant.

## 3. Results

### 3.1. Population

Between January 2019 and December 2023, a total of 214 eyes from 190 patients with glaucoma underwent standalone PMS surgery, whereas 27 eyes from 25 patients underwent combined PMS + PCE during the same period. Twelve eyes in the PMS group were aphakic, and four eyes in the PMS group and one eye in the PMS + PCE group had no light perception at baseline; these eyes were therefore excluded. In addition, among the patients who underwent standalone PMS, 62 eyes were phakic and one eye received intraoperative 5-fluorouracil; these cases were also excluded from analysis. Ultimately, the eligibility criteria were met by 26 eyes in the PMS + PCE group and by 134 pseudophakic eyes in the standalone PMS group. Before matching, baseline IOP, glaucoma type, and MMC dose differed significantly between groups. After 3:1 matching, the PMS + PCE group included 26 eyes and the PMS group included 78 eyes. Table 1 details the baseline demographic and clinical characteristics of the patients after matching.

The mean age was 64.0 ± 11.2 years in the PMS + PCE group and 64.8 ± 12.8 years in the PMS group (*p* = 0.61). After matching, no significant differences were observed for glaucoma severity, baseline IOP, or number of hypotensive medications. However, primary angle-closure glaucoma (PACG) and pseudoexfoliation glaucoma (PEXG) were overrepresented in the PMS + PCE group, whereas primary open-angle glaucoma (POAG) and secondary glaucoma were more frequent in the PMS group (*p* ≤ 0.01).

### 3.2. Efficacy

At 12 months, absolute success was observed in 6 out of 26 eyes (23.1%) in the PMS + PCE group versus 34 of 78 eyes (43.6%) in the PMS group (*p* = 0.13). Qualified success at 12 months was achieved in 14 out of 26 eyes (53.9%) in the PMS + PCE group and in 55 out of 78 eyes (70.5%) in the PMS group (*p* = 0.30). Censoring was higher in the PMS + PCE group, with 7 out of 26 eyes (26.9%) lacking 12-month data for assessment of the primary endpoint, compared with 13 out of 78 eyes (16.7%) in the PMS group.

**Figure 1:**
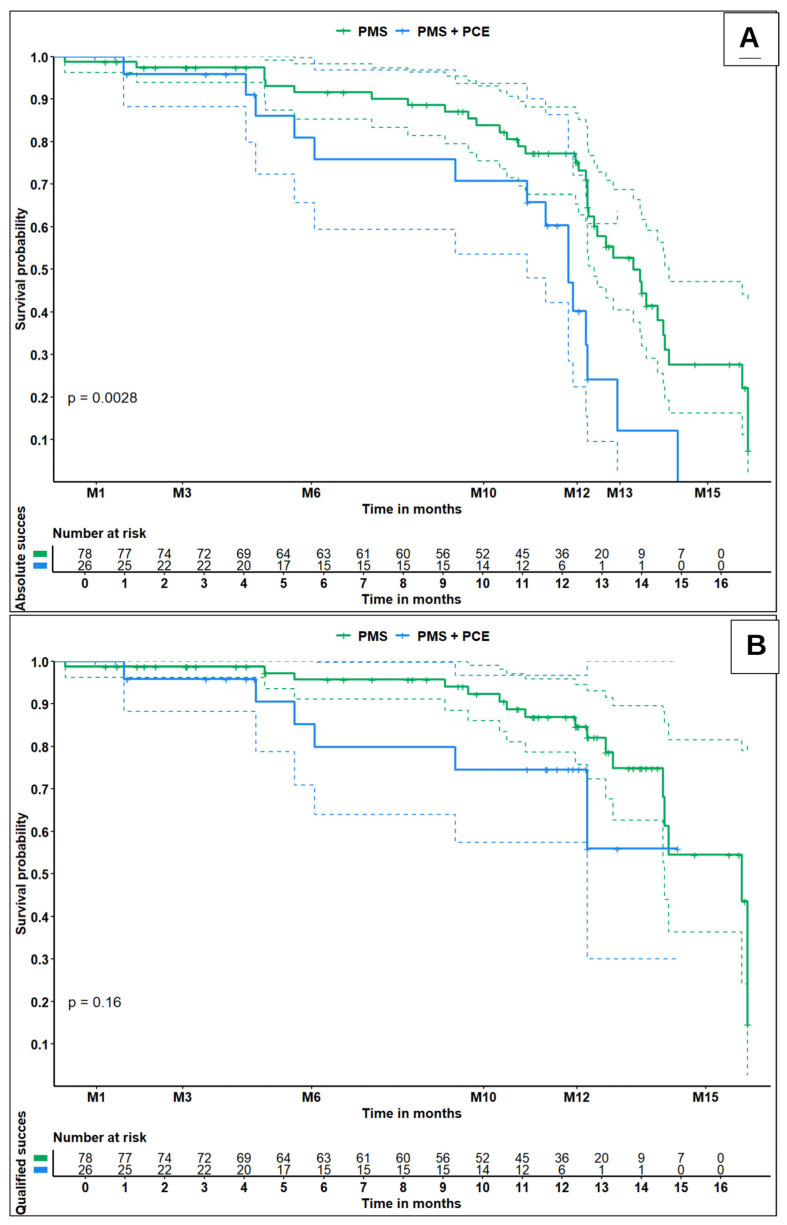
Kaplan–Meier survival curves for absolute success (**A**) and qualified success (**B**) in eyes treated with PMS alone (green line) and combined PMS + PCE (blue line). Dashed lines indicate the 95% confidence interval (95% CI). M: months; PMS: Preserflo Microshunt (standalone); PMS + PCE: Preserflo Microshunt combined with phacoemulsification. A *p*-value < 0.05 was considered statistically significant.

Kaplan–Meier survival curves showed a significant between-group difference in absolute success at 12 months (*p* = 0.0028) (Figure 1A). The 12-month cumulative survival probability for absolute success was 75.2% (95% CI, 65.3–86.7) in the PMS group versus 40.2% (95% CI, 22.4–72.1) in the PMS + PCE group. By contrast, no significant difference was observed in qualified success between groups (*p* = 0.16) (Figure 1B). The 12-month survival probabilities for qualified success were 74.5% (95% CI, 57.4–96.7) in the PMS + PCE group and 84.6% (95% CI, 75.7–94.6) in the standalone PMS group.

**Figure 2:**
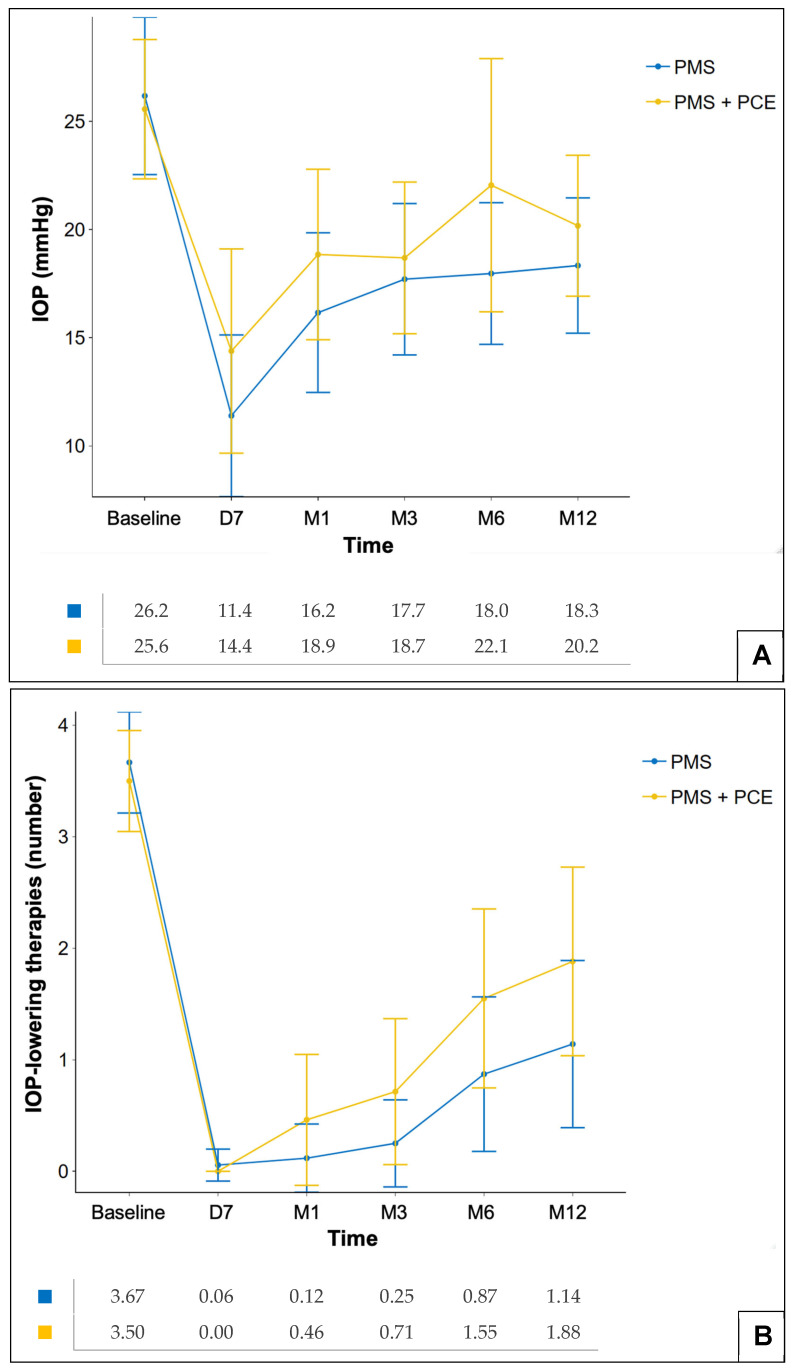
Evolution of mean IOP (**A**) and mean number of glaucoma medications (**B**) over 12 months postoperatively, excluding patients who underwent additional glaucoma surgery. Error bars represent standard deviation. Mean values are reported below each panel. D: days; M: months; PMS: Preserflo Microshunt (standalone); PMS + PCE: Preserflo Microshunt combined with phacoemulsification.

The preoperative mean IOP was 25.6 ± 6.4 mmHg in the PMS + PCE group and 26.2 ± 7.3 mmHg in the PMS group (*p* = 0.89). The postoperative IOP and number of medications on days 7 and months 1, 3, 6, and 12 are shown in Figure 2A and Figure 2B respectively. At 12 months, mean IOP was 18.3 ± 6.3 mmHg in the PMS group and 20.2 ± 6.5 mmHg in the PMS + PCE group, with no significant between-group difference (*p* = 0.40). This corresponded to an absolute reduction of −7.7 ± 7.8 mmHg (−25.7%) from baseline in the PMS group and −5.1 ± 8.2 mmHg (−17.2%) in the PMS + PCE group (*p* = 0.34). For the number of hypotensive medications, no significant difference was detected between groups at 12 months. The mean number of medications at 12 months was 1.1 ± 1.5 in the PMS group, which is a reduction of −2.5 ± 1.6 (−68.7%) from baseline, and 1.9 ± 1.7 in the PMS + PCE group, which is a reduction of −1.7 ± 1.7 (−46.7%) (*p* = 0.08).

### 3.3. Rates of Needling, Bleb Revision and Reintervention

At least one needling was performed in 26.9% of PMS + PCE eyes and in 23.1% of PMS eyes (*p* = 0.79). Among eyes requiring needling, the mean number of needlings per eye was 1.71 ± 1.11 in the combined group versus 1.33 ± 0.69 in the standalone group (*p* = 0.34). Bleb revision rates did not differ significantly between groups (*p* = 0.62). However, the mean time to first bleb revision was significantly shorter in the PMS + PCE (61.2 ± 41.9 days) than in the PMS group (152.7 ± 109.2 days; *p* = 0.031) (Table 2). Similarly, the mean time to first needling was shorter in the PMS + PCE (41.3 ± 59.5 days) compared to the PMS group (94.1 ± 73.9 days; *p* = 0.015). Most bleb revisions in the PMS + PCE group occurred within the first 3 postoperative months (83.3%, 5/6) versus 34.8% (8/23) in the PMS group (*p* = 0.064). Finally, no significant between-group difference was found in the 1-year reoperation rate for glaucoma between groups (*p* = 0.33).

### 3.4. Safety

Postoperative complications indicated broadly comparable safety profiles between the two groups (Table 2). Among early events, transient hypotony and choroidal detachment occurred at similar frequencies in both groups (2 out of 26 eyes in the PMS + PCE group versus 6 out of 78 eyes in the PMS group; 7.7% each; *p* = 1). Other early events—including hyphema, hematocornea, tube obstruction, and bleb leak—were infrequent, with no significant between-group differences. For late complications, rates were similarly low and comparable: no chronic hypotony, cystoid macular edema, or tube exposure/extrusion was reported in the PMS + PCE group, whereas isolated cases occurred in the PMS group (*p* > 0.05). Conversely, tube–endothelium touch was observed only once in the PMS + PCE group (*p* = 0.25). A decrease in BCVA of ≥2 lines (logMAR) was more frequent in the PMS group (21.8%) than in the PMS + PCE group (15.4%), without reaching statistical significance (*p* = 0.58). All other adverse events (peripheral anterior synechiae, uveitis, endophthalmitis/blebitis, diplopia, and malignant glaucoma) were rare or absent in both groups.

## 4. Discussion

This matched retrospective comparative study evaluated the efficacy and safety of the PMS implanted either as a standalone procedure in pseudophakic eyes or in combination with PCE in patients with glaucoma across varying stages of severity. At 12 months, both standalone and combined PMS were associated with significant reductions in IOP and in the number of hypotensive medications, with no major between-group differences in surgical success. However, the standalone PMS group showed a higher probability of achieving absolute success than the combined group, although this difference did not reach statistical significance. Safety profiles were comparable between procedures, with a low incidence of serious adverse events in both surgical modalities. Matching on key prognostic factors—sex, age, glaucoma severity, preoperative IOP, and prior glaucoma surgeries—helped mitigate confounding and improved comparability between groups.

A notable finding in our study is the apparent discrepancy between the 12-month point estimates and the Kaplan–Meier survival analysis for absolute success. Although the difference in absolute success rates at 12 months did not reach statistical significance (*p* = 0.13), the survival analysis revealed a significantly higher cumulative probability of success in the standalone group (*p* = 0.0028). This distinction is explained by the fact that Kaplan–Meier analysis accounts for censored data and, more importantly, incorporates eyes that failed early in the postoperative period. Because the combined group experienced more rapid bleb fibrosis and earlier requirements for intervention, these failure events are more accurately captured by time-to-event dynamics than by fixed time-point comparisons. Overall, these data suggest that patients undergoing standalone PMS implantation have a significantly higher probability of achieving and maintaining absolute, medication-free success than those undergoing the combined procedure.

These findings are consistent with the two prior comparative studies addressing this question. In a prospective study, Fili et al. found no significant difference in qualified success at 12 months (IOP < 18 mmHg and reduction > 20%) but observed a significantly higher absolute success rate using a stricter definition (IOP < 15 mmHg and reduction > 30% without medications) in the standalone PMS group (80% versus 60% in PMS + PCE; *p* = 0.022) [16]. Similarly, Martinez-de-la-Casa et al. reported no significant between-group differences in surgical success using thresholds of IOP < 18 mmHg and at least a 20% reduction, whether without medications (absolute success: 52% PMS + PCE versus 69% PMS) or with medications (qualified success: 78% PMS + PCE versus 86% PMS; *p* > 0.05) [17]. In our study, the primary success threshold of ≤21 mmHg was chosen in accordance with World Glaucoma Association guidelines [23]. While some trials utilize stricter criteria, this threshold remains a standard benchmark in real-world studies involving heterogeneous and often severe glaucoma populations. In such cases, surgical success is not defined solely by an absolute numerical value but by achieving a stable, individualized target pressure while significantly lowering the dependency on hypotensive medications.

In our cohort, we likewise observed no between-group differences in IOP reduction or medication burden between standalone PMS and PMS combined with PCE, which is in agreement with Martinez-de-la-Casa et al. and Fili et al. [16,17]. Notably, the magnitude of IOP reduction in our study was smaller than that reported in other publications [24,25]. For example, Battle et al. reported a 55.0% reduction at 1 year relative to baseline, whereas we observed a 25.7% reduction in the standalone PMS cohort [24]. The trial enrolled only POAG eyes without prior history of glaucoma surgery and used MMC 0.4 mg/mL, which likely explains the discrepancy relative to our more heterogeneous cohort with predominantly severe glaucoma, frequent prior glaucoma surgeries and use of MMC 0.2 mg/mL. Indeed, randomized trials comparing PMS to trabeculectomy have confirmed the superiority of trabeculectomy as a first-line procedure [7], and French recommendations advocate PMS as a second-line option after failure of a first filtering surgery or as a first-line option in patients at high risk of failure [26].

The PMS safety profile appeared similar between the standalone and combined procedures in our study. Serious adverse events were rare, with early transient hypotony and choroidal detachment each occurring in 7.7% of eyes in both groups. A decrease in visual acuity of at least two lines was observed in both groups (*p* = 0.58). These occurrences were largely related to early, transient postoperative complications such as hypotony and choroidal detachment, which are known risks associated with filtering procedures. As these visual acuity changes were most often temporary and balanced between the two surgical groups, they do not appear to be specifically driven by the choice of a standalone or combined approach. Nevertheless, this finding highlights the need for careful monitoring of early intraocular pressure fluctuations to preserve visual function.

The need for needling or bleb revision was comparable regardless of procedure type. However, the mean latency to bleb revision was shorter in the combined group than in the standalone PMS group, suggesting earlier bleb fibrosis following combined surgery. Several reports in the filtering-surgery literature have described accelerated conjunctival fibrosis related to the pro-inflammatory response triggered by cataract surgery [27,28,29,30]. Siriwardena et al. demonstrated persistent anterior chamber inflammation for at least three months after cataract surgery, even in uncomplicated cases [31]. Furthermore, ultrasound energy and the release of lens material during PCE can disrupt the blood–aqueous barrier and promote infiltration of pro-inflammatory cytokines (e.g., IL-8, MCP-1, and TNF-α) into the filtration bleb, potentially persisting up to three months and contributing to greater conjunctival fibrosis than in the absence of PCE [32]. This pro-inflammatory context may therefore increase the risk of PMS failure, helping to explain both the earlier bleb revisions observed in combined cases and the Kaplan–Meier findings for absolute success. In their subgroup analysis, Schlenker et al. reported similar findings for the combined PMS subgroup, with a lower rate of absolute success (defined as an intraocular pressure between 6 and 17 mmHg without medication) than with standalone implantation. The rate of qualified success (using the same IOP criteria, but allowing glaucoma medication) was broadly similar between the two groups. Likewise, the combined procedure was associated with a higher needling rate (23.3% vs. 6.8% in the standalone PMS group) in non-refractory glaucoma eyes, suggesting earlier fibrosis in the combined group. A similar phenomenon has been described for combined Xen^®^ Gel Stent procedures (AbbVie, Chicago, IL, USA) [33], with meta-analyses reporting a transient reduction in efficacy when combined with cataract surgery, likely reflecting heightened postoperative inflammation and a higher risk of mechanical stent obstruction. Importantly, these differences tend to attenuate after approximately six months, presumably because of progressive bleb remodeling and resolution of inflammation [34,35]. Wider et al. further reported that combined Xen^®^ + PCE is associated with a short-term increased risk of reoperation compared with standalone Xen^®^ [36]. It should be noted that our study did not provide objective measures of postoperative inflammation, such as laser flare photometry, nor did it record intraoperative parameters such as phacoemulsification energy or surgical duration. Therefore, the link between cataract surgery and accelerated bleb fibrosis remains a clinical hypothesis. Nevertheless, this interpretation is consistent with previous research.

To minimize any possible confounding from previous cataract surgery, only pseudophakic patients who had undergone phacoemulsification at least 6 months before the filtering surgery were included in the PMS-alone group. Furthermore, it is important to acknowledge that our study did not evaluate a stepwise surgical strategy, in which cataract surgery is performed first and glaucoma surgery only if subsequently required. This approach represents an important and commonly adopted clinical strategy, particularly because phacoemulsification itself has a recognized IOP-lowering effect even in POAG [37]. Simultaneous PMS and PCE should therefore be viewed as one feasible surgical option for patients requiring concurrent management of both conditions, rather than as a definitively superior approach to sequential treatment. Future research comparing these two strategies would help to better isolate the specific contribution of each procedure and refine the indications for combined versus sequential surgery in clinical practice.

We did not report functional or structural data, such as visual field progression or retinal nerve fiber layer (RNFL) thickness. Instead, we used IOP and medication burden as surrogate markers of glaucoma stability. From a methodological standpoint, comparing IOP and medication counts in a retrospective study is more robust than analyzing visual fields or RNFL, which are often subject to device heterogeneity and a lack of standardized protocols. These metrics provide objective, standardized data that allow for a reliable comparison.

This study has several limitations. First, it is a retrospective analysis with a small sample size, which limits its statistical power and generalizability. Second, the lack of randomization may have introduced a selection bias, given that a standalone procedure is mainly indicated for uncontrolled glaucoma, whereas combined surgery can be performed in patients with moderate to advanced glaucoma—including those with controlled intraocular pressure—who undergo cataract surgery to prevent postoperative IOP spikes. These potential biases should, however, be counterbalanced by the fact that patients in both groups were matched on baseline characteristics. Nevertheless, despite matching the cohorts on five key variables, residual confounding persists because of the observational and retrospective design of the study. Specifically, imbalances in the distribution of MMC concentration and glaucoma subtypes remained between groups. Given the relatively small sample size of the combined group (*n* = 26), an adjusted multivariable analysis was not performed, as it would have lacked the statistical power required to yield reliable results. Variability in MMC concentration constitutes a potential source of bias that may influence effectiveness; most patients underwent surgery before recent recommendations favoring 0.4 mg/mL and therefore received MMC 0.2 mg/mL [38]. As mentioned above, the imbalance in glaucoma subtypes between the two groups represents a further limitation. PEXG, which is overrepresented in the combined group, is traditionally associated with a more aggressive clinical course and a higher risk of surgical failure [39,40,41]. This could bias our results toward an underestimation of the efficacy of the combined procedure. Conversely, the higher prevalence of PACG in the same group introduces an opposing bias: in PACG, phacoemulsification alone significantly modifies the anterior chamber anatomy and provides a substantial hypotensive effect, which could lead to an overestimation of the contribution of the PMS implant itself to IOP control in the combined cohort. Nonetheless, the heterogeneity of glaucoma subtypes (POAG, PACG, PEXG, secondary, etc.) mirrors real-world clinical practice and supports broader applicability to routine glaucoma care. In the present study, bleb needling and surgical revisions were not categorized as therapeutic failures, a choice that aligns with the methodology of most major trials on subconjunctival drainage devices. These maneuvers are generally regarded as adjustments needed to modulate the wound-healing process and maintain bleb patency rather than as a loss of surgical efficacy. However, it is important to acknowledge that excluding these events may lead to a more optimistic interpretation of success. The earlier requirement for bleb maintenance in the combined group reinforces the hypothesis that phacoemulsification triggers a more rapid pro-fibrotic response. Consequently, although the long-term IOP-lowering potential remains comparable, combined procedures may require more intensive and proactive monitoring during the early postoperative period to manage this accelerated fibrotic response. Additionally, specific intraoperative data related to the phacoemulsification component of the combined procedures—such as surgical duration and phacoemulsification energy—were not available for analysis. Although all surgeries followed a standardized protocol, the lack of objective measures of surgical intensity limits our ability to precisely quantify the inflammatory burden contributed by the cataract surgery itself. Another limitation is the nontrivial proportion of patients lost to follow-up, especially in the combined group. While some of these patients may have been transferred to community ophthalmologists because of satisfactory clinical status, assuming that these cases represent successful outcomes remains speculative. This attrition may therefore introduce a bias in the 12-month report of success rates. Finally, including both eyes as independent units without adjusting for inter-eye correlation is a statistical limitation. Although bilateral cases were infrequent in our cohort (12.6% in the PMS group and 8.0% in the PMS + PCE group), this limitation may affect the precision of our estimates. Future studies should use models such as generalized estimating equations (GEEs) to account for inter-eye dependencies.

## 5. Conclusions

In conclusion, this retrospective case–control study suggests that both standalone and combined PMS implantation can lead to significant reductions in IOP and medication dependency in patients with glaucoma. Although no statistically significant differences in overall success were detected between the two approaches in our matched cohort, the standalone procedure was associated with a significantly higher probability of achieving absolute success. In addition, the shorter latency to bleb revision in the combined group highlights the potential impact of phacoemulsification-induced inflammation on the filtration site. Given the inherent confounding factors associated with the retrospective nature of this study and the limited sample size, these findings do not allow a definitive conclusion to be drawn regarding the equivalence or superiority of either strategy. Nevertheless, the combination of PMS and PCE remains a reasonable surgical option for patients requiring concurrent management of cataracts and progressive glaucoma, provided that the therapeutic strategy is carefully individualized and guided by patient-specific clinical characteristics. Prospective randomized trials are warranted to further clarify the optimal surgical sequence in this population.

## Figures and Tables

**Table 1:** Socio-demographic and clinical characteristics of patients.

Characteristics	PMS + PCE	PMS
Number (*n*)	26	78
Age (years), mean (SD)	64.0 (11.2)	64.8 (12.8)
Sex, *n* (%)		
*Female*	9 (34.6)	28 (35.9)
*Male*	17 (65.4)	50 (64.1)
Visual field MD (dB), mean (SD)	17.3 (8.9)	15.1 (7.4)
Glaucoma severity, *n* (%)		
*Early* (MD ≤ 6)	4 (15.4)	9 (11.5)
*Moderate* (6 < MD ≤ 12)	3 (11.5)	8 (10.3)
*Severe* (MD > 12)	19 (73.1)	61 (78.2)
Cup/disc ratio, mean (SD)	0.89 (0.15)	0.84 (0.19)
Type of glaucoma, *n* (%)		
*POAG*	10 (38.5)	42 (53.8)
*PACG*	5 (19.2)	1 (1.3)
*PEXG*	7 (26.9)	2 (2.6)
*Juvenile and congenital glaucoma*	1 (3.8)	2 (2.6)
*Others (uveitic, neovascular, etc.)*	3 (11.5)	31 (39.7)
Baseline BCVA (logMAR), mean (SD)	0.67 (0.83)	0.58 (0.73)
Baseline IOP (mmHg), mean (SD)	25.6 (6.4)	26.2 (7.3)
Baseline hypotensive medications, mean (SD)	3.5 (0.9)	3.7 (0.9)
Prior SLT, *n* (%)	7 (26.9)	19 (24.4)
Number of prior SLT, mean (SD)	1.29 (0.76)	1.26 (0.45)
Number of prior glaucoma surgeries, mean (SD)	1.15 (1.35)	1.41 (1.21)
Previous glaucoma surgery, *n* (%)		
*Trabeculectomy*	37 (47.4)	11 (42.3)
*Non-penetrating deep sclerectomy*	33 (42.3)	8 (30.8)
*Xen Gel 45^®^*	9 (11.5)	0 (0)
*Glaucoma drainage device*	1 (1.3)	0 (0)
Prior diode laser cycloablation, *n* (%)	11 (14.1)	6 (23.1)
MMC concentration		
*0.2 mg/mL*	21 (80.8)	74 (94.9)
*0.4 mg/mL*	5 (19.2)	4 (5.1)
Follow up (months), mean (SD)	8.3 (4.4)	10.4 (4.2)

BCVA: best corrected visual acuity; dB: decibels; IOP: intraocular pressure; MD: mean deviation on the visual field; MMC: Mitomycin C; mmHg: millimeters of mercury; *n*: number; PACG: primary angle-closure glaucoma; PEXG: pseudo exfoliative glaucoma; PMS: Preserflo Microshunt; PMS + PCE: Preserflo Microshunt + phacoemulsification; POAG: primary open-angle glaucoma; SD: standard deviation; SLT: selective laser trabeculoplasty. A value of *p* < 0.05 is considered significant.

**Table 2:** Postoperative complications in PMS + PCE versus PMS.

Postoperative Complications	PMS + PCE *n* = 26	PMS *n* = 78	*p*
**Early Postoperative complications**			
Shallow anterior chamber, *n* (%)	1 (3.9)	1 (1.3)	0.44
Choroidal detachment, *n* (%)	2 (7.7)	6 (7.7)	1
Transient hypotony *, *n* (%)	2 (7.7)	6 (7.7)	1
Occlusion tube, *n* (%)	2 (7.7)	3 (3.9)	0.6
Hyphema, *n* (%)	3 (11.5)	8 (10.3)	1
Hematocornea, *n* (%)	0 (0)	1 (1.3)	1
Seidel, *n* (%)	0 (0)	1 (1.3)	1
**Chronic postoperative complications**			
Chronic hypotony **, *n* (%)	0 (0)	0 (0)	1
Macular edema, *n* (%)	0 (0)	6 (7.7)	0.33
Tube–endothelium contact, *n* (%)	1 (3.9)	0 (0)	0.25
Endothelial decompensation, *n* (%)	0 (0)	2 (2.6)	1
Tube exposure, *n* (%)	0 (0)	2 (2.6)	1
Tube exteriorization, *n* (%)	0 (0)	2 (2.6)	1
Breakage, migration, tube cross-section, *n* (%)	0 (0)	0 (0)	1
Decrease in visual acuity ≥ 2 lines (logMAR), *n* (%)	4 (15.4)	17 (21.8)	0.58
Wipeout, *n* (%)	0 (0)	1 (1.3)	1
Phthisis bulbi, *n* (%)	0 (0)	0 (0)	1
**Other, *n* (%)**			
Development of peripheral anterior synechiae	1 (3.9)	0 (0)	0.25
Uveitis	0 (0)	1 (1.3)	1
Endophthalmitis/Blebitis	0 (0)	0 (0)	1
Diplopia	0 (0)	0 (0)	1
Malignant glaucoma	0 (0)	0 (0)	1
**Need for additional procedures**			
Needling, *n* (%)	7 (26.9)	18 (23.1)	0.79
Surgical bleb revision, *n* (%)	6 (23.1)	23 (29.5)	0.62
Time to 1st needling (days), mean (SD)	41.3 (59.5)	94.1 (73.9)	0.015
Time to 1st surgical revision (days), mean (SD)	61.2 (41.9)	152.7 (109.2)	0.031
New glaucoma surgery within a year, *n* (%)	5 (19.2)	9 (11.5)	0.33

* Transient hypotony: intraocular pressure below 6 mmHg for less than one month. ** Chronic hypotony: persistence of intraocular pressure below 5 mmHg at 2 visits 3 months apart; *n*: number; PMS: Preserflo Microshunt; PMS + PCE: Preserflo Microshunt + phacoemulsification; SD: standard deviation. A value of *p* < 0.05 is considered significant.

## Data Availability

The original contributions presented in this study are included in the article. Further inquiries can be directed to the corresponding author.

## Abbreviations

The following abbreviations are used in this manuscript:

IOP	Intraocular Pressure
BCVA	Best Corrected Visual acuity
MD	Mean Deviation
MIGS	Mini Invasive Glaucoma Surgery
MMC	Mitomycin C
mmHg	Millimeters of Mercury
POAG	Primary Open-Angle Glaucoma
PEXG	Pseudoexfoliative Glaucoma
PACG	Primary Angle-Closure Glaucoma
PMS	Preserflo MicroShunt
PCE	Phacoemulsification
SLT	Selective Laser Trabeculoplasty
5-FU	5-Fluorouroacile
